# Species diversity of edible mushrooms IV: Morphology and phylogenetic analysis reveal two new species of *Lyophyllum* (*Lyophyllaceae*, *Basidiomycota*) from Yunnan Province, Southwestern China

**DOI:** 10.3897/mycokeys.136.193238

**Published:** 2026-06-26

**Authors:** Rui-Yu Li, Yuan Luo, Duan-Fen Zhao, Ye-Ting Li, Han-Bing Song, Hong-Wei Shen, Zong-Long Luo, Song-Ming Tang

**Affiliations:** 1 College of Agriculture and Biological Science, Dali University, Dali 671003, Yunnan, China College of Agriculture and Biological Science, Dali University Dali China; 2 Co-Innovation Center for Cangshan Mountain and Erhai Lake Integrated Protection and Green Development of Yunnan Province, Dali University, Dali 671003, Yunnan, China Co-Innovation Center for Cangshan Mountain and Erhai Lake Integrated Protection and Green Development of Yunnan Province, Dali University Dali China

**Keywords:** Morphology, multigene phylogeny, taxonomy, two novel taxa

## Abstract

*Lyophyllum* species holds significant economic and medicinal value. During an investigation of macrofungi in southwestern China, seven *Lyophyllum* specimens were collected. Detailed morphological examinations, together with multi-locus phylogenetic analyses based on internal transcribed spacer (ITS), nuclear ribosomal large subunit (nrLSU), RNA polymerase II second largest subunit (*rpb*2), and translation elongation factor 1-alpha (*tef*1-α) sequence data, revealed two previously undescribed species, *Lyophyllum hemigaleatum* and *L. pseudorrhizum*. *Lyophyllum hemigaleatum* has an orange and hemispherical pileus (1.4–1.8 cm), inflexed of margin; pileus context white, fleshy, and turns slightly desaturated orange when injured; basidiospores broadly to subbroadly (av. 7.36 ± 0.65 × 5.10 ± 0.44 μm); in multi-locus phylogenetic analysis, *Lyophyllum hemigaleatum* is closely related to *L. semitale*, *L. semitale* was originally described from Sweden, has a larger pileus (2.5 cm vs. 1.4–1.8 cm) and a white stipe; its most distinctive feature is a distinctly rooted stipe and saprophytic on wood, these pieces of evidence support our tentative identification of *L. hemigaleatum* as new species. *Lyophyllum pseudorrhizum* is characterised by a fleshy pileus (1.2–2.8 cm wide) that is greyish orange, hemispherical when young, becoming plano-convex or convex with age and with an inflexed margin; pileus context is white and unchanged in colour when injured; stipe base aggregates to form pseudorrhiza; basidiospores subglobose to broadly ellipsoid (av. 5.2 ± 0.42 × 4.8 ± 0.34 μm). Descriptions, illustrations and phylogenetic analysis results of the new species are provided. In addition, the new species are compared with closely related taxa.

## Introduction

*Lyophyllum* P. Karst. is the type genus of the family *Lyophyllaceae* Jülich., established based on the type species *L. leucophaeatum* (P. Karst.) P. Karst. (Karsten 1881). Currently, more than 70 species have been described worldwide (He et al. 2019; Xu et al. 2021; Arana-Gabriel et al. 2025; Tang et al. 2025a). Species of this genus are characterised by basidiomata that grow solitary, scattered or clustered; saprophytic or symbiotic; pileus surface mostly smooth, rarely floccose; stipes are cylindrical or enlarged toward the base, with some species possessing a tuberous stem base; and basidiospores are globose, ellipsoid, or broadly fusiform, in addition, some species discolour when bruised (Tang et al. 2025a).

The taxonomic study of the *Lyophyllum* has evolved through distinct phases, transitioning from morphology-based classifications to modern phylogenetic integration (Wang et al. 2013). Karsten (1881) established the initial taxonomic framework, including sect. *Lyophyllum* P. Karst. And its subordinate subsect. *Lyophyllum* P. Karst. In subsequent morphological classifications, Singer (1986) further divided *Lyophyllum* into three sections based on two key macroscopic traits: the growth habits and bruising reactions of the basidiomata. Specifically, sect. *Lyophyllum* includes species whose basidiomata turn black when injured; sect. *Difformia* Singer comprises caespitose species that do not discolour when bruised; and sect. *Tephrophana* Singer contains species with a collybioid morphology. To address the limitation of sect. *Difformia* (which originally only included caespitose species), Consiglio and Contu (2002) established subsect. *Lanzoniana* Consiglio & Contu, characterised by non-caespitose basidiomata with unchanging flesh. All these classifications relied heavily on macroscopic traits. However, these systems proved unstable because macroscopic characteristics are often plastic (influenced by environmental factors) and subjective (varying among researchers). In response to this instability, Gerhardt (1982) established the new subgenus *Lyophyllopsis* Ew. Gerhardt with *Lyophyllum* to accommodate the newly described species *L. incarnatobrunneum* Ew. Gerhardt, a species distinct from other *Lyophyllum* members due to the absence of clamp connections. Later, Bon (1994) elevated this subgenus to generic rank, introducing the new genus *Gerhardtia* Bon. More recently, Picillo et al. (2014) recommended retaining ambiguous groups provisionally within *Lyophyllum* sensu lato until further phylogenetic evidence becomes available.

With the advent of molecular systematics, traditional morphology-based classifications of *Lyophyllum* have been extensively re-evaluated. Moncalvo et al. (1993) used ITS sequences to demonstrate the polyphyly of sect. *Difformia*; this finding was subsequently confirmed by Hofstetter et al. (2002), who further showed through multi-gene analyses that *Lyophyllum* along with its traditional sections is polyphyletic. Phylogenetically, a key contradiction emerged: the original type species, *L. leucophaeatum* was shown to cluster with *Calocybe* sensu lato (including its type species *C. gambosa* (Fr.) Donk) rather than with other species traditionally assigned to *Lyophyllum*. To resolve this conflict and maintain nomenclatural stability, *Lyophyllum* was conserved with *L. semitale* (Fr.) Kühner designated as the conserved type species (Redhead et al. 2006). Despite these molecular-driven revisions, the currently recognized infrageneric classification system is still based on Singer (1986) division of *Lyophyllum* into three sections, which is primarily based on the growth habits and bruising reactions of the basidiomata. However, the ongoing discovery of new species has continued to fuel debate over the utility of morphological characteristics in infrageneric delimitation (Colucci and Galli 2010; Dähncke et al. 2010, 2011; Vizzini et al. 2010; Contu et al. 2011). Current consensus in the field emphasizes integrating multi-locus phylogenetic data with refined morphological examination to reconstruct a natural classification. At the same time, it acknowledges that the taxonomic positions of several groups remain unresolved pending more extensive molecular sampling.

As of today, 27 species have been recorded in China, of which 14 species of *Lyophyllum* were originally described in China, *L. atrofuscum* S.W. Wei, Q. Wang & Yu Li, *L. aurantiacum* R.Y. Li, Z.L. Luo & S.M. Tang, *L. bulborhizum* S.M. Tang & S.H. Li, *L. deqinense* Y.H. Ma, W.M. Chen & Y.C. Zhao, *L. edulis* S.M. Tang & Shu H. Li, *L. heimogu* Shu H. Li, *L. lixivium* S.M. Tang & Shu H. Li, *L. nigrum* S.M. Tang & S.H. Li, *L. pallidofumosum* Y.H. Ma, W.M. Chen & Y.C. Zhao, *L. rhombisporum* S.H. Li & Y.C. Zhao, *L. sinense* S.M. Tang & Shu H. Li, *L. subdecastes* S.W. Wei, Q. Wang & Yu Li, *L. yiqunyang* Shu H. Li (Li et al. 2023; Melis et al. 2023; Wei et al. 2023; Li et al. 2025; Tang et al. 2025a). Most of these species were established based on specimens from Yunnan Province, with Yunnan serving as their type locality. From 2023 to 2024, we conducted field surveys on macrofungi in the sub-plateau mountainous climate region of Yunnan Province, China, an area noted for its diverse vegetation and favourable climatic conditions that support high fungal diversity. Through a comprehensive survey of *Lyophyllum* and using both morphological characteristics and multi-locus phylogenetic analyses (ITS, LSU, *rpb*2, and *tef1*-α), we discovered two novel *Lyophyllum* species, which are formally described in this study.

## Materials and methods

### Specimens collected

The specimens were collected in Yunnan province, China. In the field, *in situ* photographs of fresh basidiomata and macroscopic data on the basidiomata, along with details of the collection site, date, habitat, substrate, elevation, and geographical coordinates, were recorded. The basidiomata were placed in plastic mushroom collection boxes and taken to the mycology laboratory at Dali University. Fresh specimens were then dried at 55 °C in a food dryer and stored in sealed plastic bags. The specimens are deposited at the Herbarium of Cryptogams, Kunming Institute of Botany, Chinese Academy of Sciences (HKAS) and the Herbarium of Dali University.

### Morphological studies

Macromorphology was documented from fresh basidiomata; colours were matched using the Color Hexa website (https://www.colorhexa.com/), and terminology followed Bas (Bas 1990). For micromorphology, dried material was sectioned and mounted in 5% KOH; when observation was unclear, a 1% Congo Red staining solution was used to assist in examination, and the specimen was examined with a Nikon ECLIPSE Ni-U microscope equipped with a DS-Ri2 digital camera. At 1000× magnification, 20 basidia and 50 basidiospores per collection were measured. The notation [x/y/z] indicates that measurements were taken from ‘x’ basidiospores from ‘y’ basidiomata in ‘z’ collections. Basidiospore dimensions are presented as ‘(a) b–n–c (d)’, where ‘a’ and ‘d’ represent the minimum and maximum values of all measurements, respectively, ‘b–c’ represents the range of 95% of the measured basidiospores, ‘n’ refers to the average measurement, ‘Q’ is the length/width ratio of basidiospores, and ‘Q_m_’ is the average Q value ± standard deviation of all basidiospores (Tang et al. 2025b).

### DNA extraction, PCR amplification and sequencing

DNA was extracted from dry specimens using the EZup Fungi Genomic DNA Extraction Kit (Sangon Biotech, Shanghai, China) according to the manufacturer’s protocol. For PCR amplification, the PCR volume contained 1 µL of each primer, 1 µL of extracted DNA, 9.5 µL of ddH2O, and 12.5 µL of 2 × EasyTaq PCR SuperMix (Sangon Biotechnology Co., Kunming, China), the ITS regions were amplified with primer pairs ITS1 and ITS4 (White et al. 1990), LR0R and LR5 for LSU (Vilgalys and Hester 1990), 6F and 7.1R for *rpb*2 (Liu et al. 1999), 983F and 2218R for *tef*1-α (Rehner and Buckley 2005). PCR was conducted using a C1000 Thermal Cycler (Bio-Rad, Beijing, China) with the following conditions: for ITS and LSU, an initial denaturation at 94 °C for 5 minutes, 35 cycles of denaturation at 94 °C for 30 seconds, annealing at 48 °C for 30 seconds, extension at 72 °C for 90 seconds, and a final extension at 72 °C for 10 minutes (White et al. 1990); for *rpb*2 and *tef*1-α, an initial denaturation at 95 °C for 5 minutes, 35 cycles of denaturation at 95 °C for 60 seconds (*rpb*2) or 90 seconds (*tef*1-α), annealing at 50 °C for 90 seconds, extension at 72 °C for 60 seconds, and a final extension at 72 °C for 10 minutes. The PCR products were sent to Sangon Biotech (Kunming, Yunnan, China) for Sanger sequencing.

### Sequence alignment and phylogenetic analyses

The newly generated sequences in this study have been deposited in GenBank (https://www.ncbi.nlm.nih.gov/genbank/), along with other similar sequences downloaded from the NCBI (https://www.ncbi.nlm.nih.gov/) datasets, *Calocybe carnea* Kühner, *C. persicolor* (Fr.) Singer and *C. ionides* (Bull.) Kühner were selected as the outgroup taxa (Hofstetter et al. 2002). Clusters of closely related sequences were analyzed for comparison (see Table 1). Gene sequences were aligned using MAFFT v.7 (Katoh and Standley 2013) and manually trimmed and quality-checked with BioEdit v.7 (Hall 2011). Subsequently, maximum likelihood (ML) analysis was performed for each gene using RAxML-HPC2 v.8.2.3 (Stamatakis 2014) on CIPRES (Miller et al. 2010), under the GTR+G+I model with 1,000 rapid bootstrap replicates. The four single-gene alignments were concatenated into a combined dataset using Sequence Matrix (Vaidya et al. 2011).

**Table 1.** Names, voucher numbers, origin and GenBank accession numbers of the sequences used in this study. Newly generated sequences are shown in bold. “*” following a species name indicates that the specimen is the type of that species, “–” refers to sequence data not available.

**Taxon name**	**Voucher numbers**	**Origin**	**GenBank**			
			**ITS**	**LSU**	**rpb2**	***tef*1-α**
*Calocybe carnea*	CBS 552.50	Switzerland	AF357028	AF223178	DQ367432	DQ367425
*C. persicolor*	IE-BSG-HC 80/99	Switzerland	AF357026	AF223176	EF420993	EF421059
*C. ionides*	IE-BSG-HC 77/133	Switzerland	AF357029	AF223179	EF420991	EF421057
*Lyophyllum ambustum*	CBS 452.87	Switzerland	AF357057	AF223216	–	–
*L. anthracophilum*	IE-BSG-HC 79/132	Switzerland	AF357055	AF223212	–	–
*L. atratum*	CBS 709.87	Switzerland	AF357053	AF223210	–	–
*L. atrofuscum*	HMJAU 63456*	China	OP605494	OP605514	–	–
	HMJAU 63461	China	OP605493	–	–	–
*L. aurantiacum*	HKAS 147010*	China	PV569793	PV569819	–	–
	HKAS 147011	China	PV569796	PV569822	–	–
*L. bulborhizum*	L5083*	China	PP406876	PQ471271	PQ523769	PQ533687
	L5092	China	PP406877	PQ471272	PQ523770	PQ533688
	L5093	China	PP406878	PQ471273	–	–
*L. caerulescens*	IE-BSG-HC80/140	Switzerland	AF357052	AF223209	EF421000	EF421066
	V. 15759	USA	JF908339	–	–	–
*L. crassifolium*	V. 5077	Italy	JF908331	–	–	–
*L. cryptofumosum* (ined.)	ABI C04207	Vietnam	LC917226	LC917227	–	–
*L. decastes*	IE-BSG-JM87/16	China	AF357059	AF042583	DQ367433	DQ367426
	Ld 418	China	HM119485	–	–	–
*L. deliberatum*	V. 15032	Slovenia	JF908338	–	–	–
*L. deqinense*	YAAS M6948	China	OQ418117	–	–	–
	YAAS M6949*	China	OQ418116	–	–	–
*L. edulis*	HKAS 135644*	China	PQ471283	PQ471265	PQ523777	PQ533681
	HKAS 135645	China	PQ471284	PQ471266	PQ523776	PQ533682
*L. favrei*	IE-BSG-HC96cp4	-	EF421102	AF223184	EF420990	EF421056
	V. 6334	Italy	JF908333	–	–	–
*L. fumosum*	LAS00-144	Sweden	HM572541	–	–	–
	V. 16077	Italy	JF908340	–	–	–
*L. gangraenosum*	V. 12332	Italy	JF908335	–	–	–
*L. heimogu*	L3026*	China	KY434100	PQ471278	PQ523782	PQ533689
	L3033	China	KY434101	PQ471276	PQ523783	PQ533690
	L3035	China	KY434102	PQ471277	PQ523784	PQ533691
***L. hemigaleatum***	**HKAS 150782**	**China**	**PX427283**	**PX427347**	**PX767157**	**PX767159**
	**HKAS 150788**	**China**	**PX427284**	**PX427348**	**PX767158**	**PX767160**
*L. herrerae*	CIRB H02*	Moxico	OR116956	**–**	**–**	**–**
*L. infumatum*	V. 10152	Italy	JF908334	–	–	–
*L. konradianum*	FR2013257	France	KP192569	–	–	–
*L. leucophaeatum*	IE-BSG-HAe251.97	Switzerland	AF357032	AF223202	DQ367434	DQ367427
*L. littoralis*	CA 20091130	Italy	JX280410	–	–	–
*L. lixivium*	HKAS 129929	China	OR506463	OR506579	–	–
	HKAS 129930	China	OR519870	OR519872	–	–
*L. loricatum*	01.12.09	Italy	JX280407	–	–	–
	V. 13175	USA	JF908336	–	–	–
	CA 20090202.03	Italy	JX280406	–	–	–
*L. moncalvoanum*	PDD 102581	New Zealand	KJ461912	–	–	–
	PDD 96328*	New Zealand	KJ461904	KJ461905	–	–
*L. nigrum*	L5091*	China	PP406873	PQ471273	PQ523773	PQ533692
	L5186	China	PP406874	PQ471274	PQ523774	PQ533693
	L5187	China	PP406875	PQ471275	PQ523775	PQ533694
*L. ochraceum*	IE-BSG-BSI94.cp1	Switzerland	AF357033	AF223185	–	–
	V. 537	Italy	JF908329	–	–	–
*L. pallidofumosum*	YAAS M6628*	China	ON680829	ON834576	–	–
	YAAS M6629	China	ON680830	ON834577	–	–
***L. pseudorrhizum***	HKAS 150783*	**China**	**PX427286**	**PX427350**	**PX767161**	**PX767166**
	**HKAS 150784**	**China**	**PX427285**	**PX427349**	**PX767162**	**PX767167**
	**HKAS 150785**	**China**	**PX427289**	**PX427353**	**PX767163**	**PX767168**
	**HKAS 150786**	**China**	**PX427288**	**PX427352**	**PX767164**	**PX767169**
	**HKAS 150787**	**China**	**PX427287**	**PX427351**	**PX767165**	**PX767170**
*L. rhombisporum*	L5084	China	PP406880	–	–	–
	L1762*	China	JX966307	–	–	–
	L2082	China	JX966308	–	–	–
	L5010	China	PP406879	–	–	–
*L. semitale*	IE-BSG-HC 85/13	Switzerland	AF357049	AF042581	EF421002	EF421068
	EL 187-09	Sweden	HM572552	–	–	–
*L. shimeji*	L 2010512377	China	JX966311	–	–	–
	Olsen 813006	Sweden	HM572530	–	–	–
	NZ4Q 88	New Zealand	JN983985	–	–	–
	HKAS 135647	China	PQ471285	PQ471267	PQ523778	PQ533683
*L. sinense*	HKAS 144417	China	PQ471281	PQ471263	–	PQ533679
*L. subalpinarum*	HMJAU 63447*	China	OP605490	–	–	–
	HMJAU 63453	China	OP605491	–	–	–
*L. subdecastes*	HMJAU 63467*	China	OP605489	–	–	–
	HMJAU 63470	China	OP605488	–	–	–
*L. sykosporum*	IFO 30978	Japan	AF357050	AF223208	EF421003	EF421069
*L. turcicum*	KATO-2971*	Turkey	KJ158159	–	–	–
*L. yiqunyang*	L2989*	China	KY434103	PV569825	–	–
	L4206	China	KY434104	PV569826	–	–

Bayesian inference was performed using MrBayes v.3.2 (Ronquist et al. 2011). The best-fit substitution model for each partition was selected with MrModeltest v.2.3 (Nylander et al. 2004). Two independent runs of six Markov Chain Monte Carlo (MCMC) chains each were conducted for 555,000 generations, with trees sampled every 100 generations and the first 25% discarded as burn-in (Rannala and Yang 1996). The runs were terminated automatically using the stoprule command once the average standard deviation of split frequencies fell below 0.01. A clade was considered strongly supported if it received bootstrap support (BS) ≥ 75% in ML analysis and a Bayesian posterior probability (PP) ≥ 0.90. The alignment was submitted to Figshare (https://doi.org/10.6084/m9.figshare.31832938).

## Results

### Molecular phylogeny

In this study, a total of 28 new sequences were generated from seven samples, comprising seven ITS, seven LSU, seven *rpb*2, and seven *tef*1-α sequences. All sequences have been deposited in the National Center for Biotechnology Information (NCBI) database. For phylogenetic analyses, 187 sequences were utilised, including 77 ITS, 49 LSU, 30 *rpb*2, and 31 *tef*1-α sequences. These sequences were obtained from 77 samples representing 43 species, with *Calocybe* selected as the outgroup (Hofstetter et al. 2002). The concatenated dataset of four loci (ITS: 1–549 bp; LSU: 550–1,399 bp; *rpb*2: 1,400–2,465 bp; *tef*1-α: 2,466–3,250 bp) yielded a total alignment length of 3,250 bp (including gaps).

**Figure 1.**
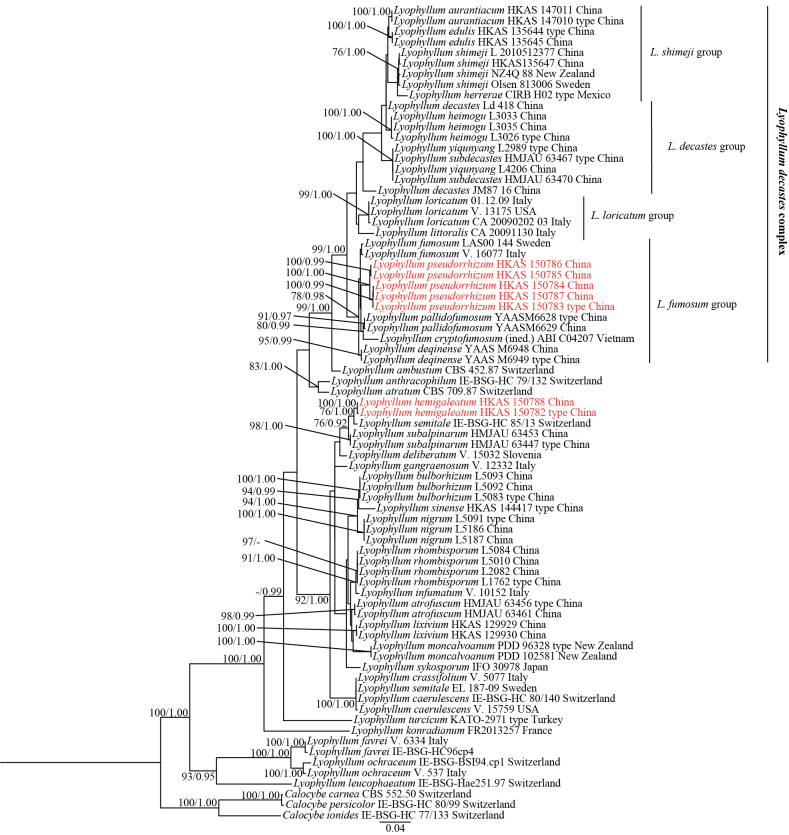
Phylogenetic relationships inferred from the concatenated sequences of ITS + LSU + *rpb*2 + *tef*1-α, presented as a strict consensus tree. Support values at nodes represent maximum likelihood bootstrap proportions (≥ 75%) and Bayesian posterior probabilities (≥ 0.90). *Calocybe ionides*, *C. carnea*, and *C. persicolor* were designated as the outgroup. Sequences obtained in this study are indicated in red.

The phylogenetic analysis of the combined dataset using RAxML resulted in a best-scoring tree with a likelihood of -16510.698700. The underlying alignment matrix, which included 1,112 distinct patterns, contained 49.54% undetermined or gapped sites. The estimated base frequencies were: A = 0.244612, C = 0.226565, G = 0.262276, T = 0.266547; substitution rates AC = 1.843088, AG = 5.846893, AT = 1.922162, CG = 1.092247, CT = 11.495549, GT = 1.000000, α = 0.273187; tree length = 1.503628. The best-fit model for Bayesian analysis of all four single-gene datasets determined using MrModeltest 2.3 was GTR+G [Lset nst = 6, rates = gamma; Prset statefreqpr = dirichlet (1, 1, 1, 1)]. The ML and BI analyses generated nearly identical tree topologies with virtually no difference in statistical support. Therefore, only the ML tree is shown (Fig. 1). Multi-locus phylogenetic analysis revealed that the seven *Lyophyllum* samples clustered into two well-supported independent clades (Fig. 1), which are described as two new species: *Lyophyllum pseudorrhizum* (78/0.98) formed a clade with *L. pallidofumosum*, *L. fumosum* (Pers.) P.D. Orton, and *L. deqinense*; whereas *L. hemigaleatum* (76/1.00) formed a sister clade with *L. semitale*.

### Taxonomy

#### Lyophyllum pseudorrhizum

Taxon classificationFungiAgaricalesLyophyllaceae

R.Y. Li & S.M. Tang sp. nov.

5B5F8803-F705-5126-B59E-31FDB0107E12

Fungal Names: FN 573141

Figs 2A–C, 3, 4

##### Chinese name.

假根离褶伞 (Jia Gen Li Zhe San).

**Figure 2.**
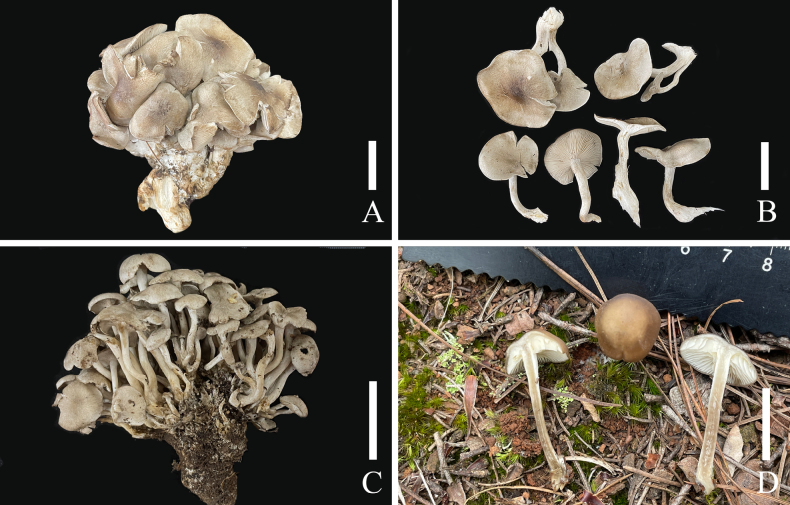
Basidiomata of *Lyophyllum*. **A–C**. *L. pseudorrhizum* (**A, B**. HKAS 150783, holotype; **C**. HKAS 150785, paratype); **D**. *L. hemigaleatum* (HKAS 150782, holotype). Scale bars: 2 cm (**A, B, D**); 5 cm (**C**).

**Figure 3.**
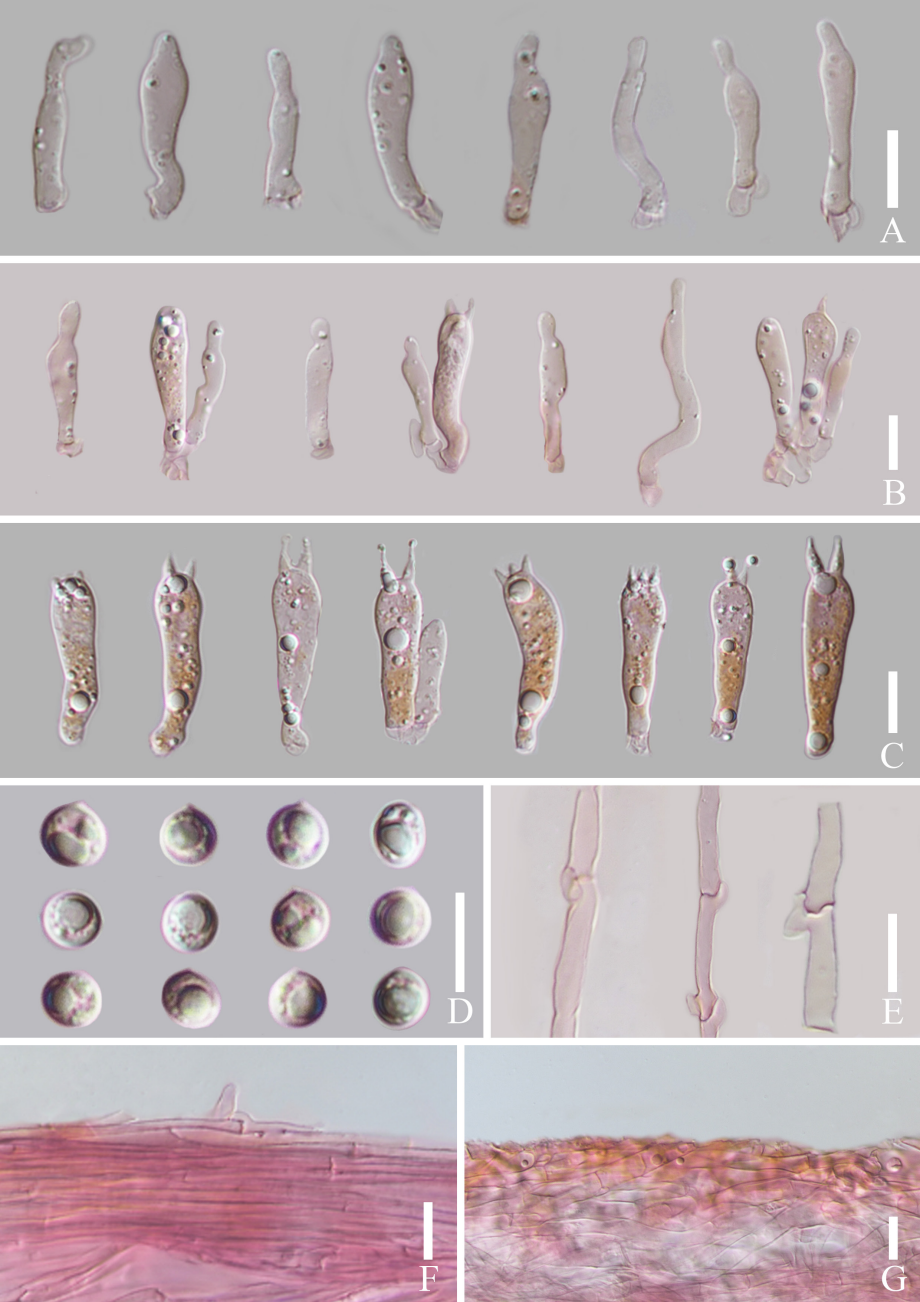
*Lyophyllum pseudorrhizum* (HKAS 150783, holotype). **A**. Pleurocystidia; **B**. Cheilocystidia; **C**. Basidia; **D**. Basidiospores; **E**. Clamp connections of the hymenophoral trama; **F**. Stipitipellis; **G**. Pileipellis. Scale bars: 10 μm (**A–G**).

**Figure 4.**
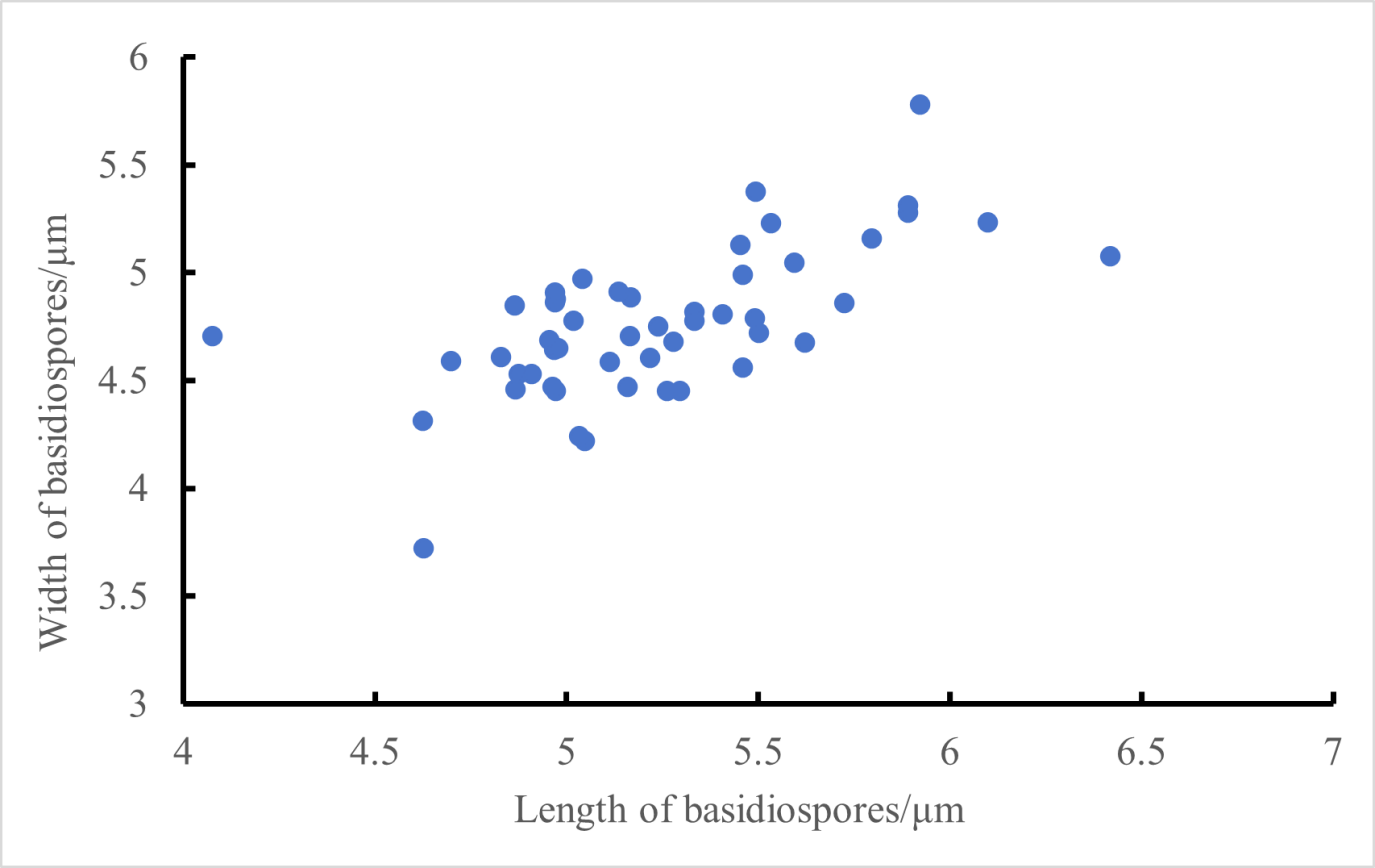
Basidiospore scatter plot of *Lyophyllum pseudorrhizum*.

##### Etymology.

The “pseudorrhizum” is derived from the prefix “*pseudo-*” (false) and “*rhiza*” (root), referring to the distinctive pseudorrhiza (false root-like structure).

##### Holotype.

China, Yunnan Province, Wuding County, elev. 2,200 m, 23 September 2023, Song-Ming Tang, L5098 (HKAS 150783!).

##### Description.

***Pileus*** 1.2–2.8 cm wide, fleshy, fragile, hemispherical when young, becoming plano-convex to convex with age, smooth on the surface, dry, greyish orange (#d6caad), with dark greyish orange floccus on the surface, slightly depressed at the centre, inflexed of margin; pileus context 0.1–0.2 cm thick, white (#ffffff), unchanging colour when injured. ***Lamellae*** moderately close together, arcuate, decurrent to deeply decurrent, narrow, 0.1–0.3 cm wide, white (#ffffff), unchanging colour when bruised, 2–3 tiers, edge even or entire. ***Stipe*** 1.8–6.8 × 0.3–0.6 cm, cylindrical, white (#ffffff) or greyish orange (#d2c8b9), smooth; stipe context white (#ffffff), solid, unchanging colour when injured, the base aggregates to form pseudorrhiza. The odour and taste were not distinctive.

***Basidiospores*** [100/2/2] (4.1–) 4.6–6.0 (–6.4) × (3.7–) 4.2–5.3 (–5.8) μm, (N = 100, Q = 1.0–1.3, Q_m_ = 1.10 ± 0.07), av. 5.2 ± 0.42 × 4.8 ± 0.34 μm, subglobose to broadly ellipsoid, hyaline, smooth, basidiospores scatter plot, see Fig. 4. ***Basidia*** 26–36 × 6–9 μm (N = 20), av. 29.7 ± 3.2 × 7.7 ± 0.7 μm, mostly 4–spored, rarely 2–spored, sterigmata 1.5–6.7 μm long, sometimes with basal clamp connections, clavate. ***Subhymenium*** thin-walled, 7–15 μm thick, composed of 2–3 layers of ovoid, subglobose, fusiform to median constriction cells, 2.7–9.6 × 1.9–4.3 μm, av. 4.7 ± 1.7 × 3.2 ± 0.6 μm. ***Hymenophoral trama*** regular, 90–110 μm wide, consisting of thin and hyaline hyphae, rarely with clamp connections; hyphal elements narrowly cylindrical, 5–10 μm wide. ***Pleurocystidia*** 17–26 × 3–6 μm, av. 22.1 ± 2.5 × 4.3 ± 1.1 μm, narrowly cylindrical or narrowly clavate, thin-walled, frequently mucronate or rostrate at the apex. ***Cheilocystidia*** 19–38 × 3–6 μm, av. 24.6 ± 4.8 × 4.1 ± 0.7 μm, narrowly cylindrical or narrowly clavate, thin-walled, frequently mucronate or rostrate on the apex. ***Pileipellis*** a subcutis composed of almost hyaline interwoven filamentous hyphae, terminal cells 3–6 μm wide, almost cylindrical to subcylindrical, occasional hyphal tips flexuous and sometimes inflate, some with clamp connections. ***Stipitipellis*** a cutis of appressed, parallel, thin-walled, hyphae 2–5 µm wide; rarely caulocystidia on the stipe, 7–42 × 2–4 µm, av. 20.8 ± 11 × 2.7 ± 0.7 µm, narrowly cylindrical or fusiform, irregular, thin-walled. ***Clamp connections*** are present at some septa.

##### Habitat.

Caespitose on soil in coniferous forest.

##### Edibility.

Edible, available in local markets.

##### Additional specimens examined.

China • Yunnan Province, Wuding County, elev. 2,190 m, 25 September 2023, Song-Ming Tang, L5198 (HKAS 150785, paratype) • *ibid*., Song-Ming Tang, L5183 (HKAS 150787) • *ibid*., Lijiang City, Yulong Naxi Autonomous County, elev. 2,470 m, 19 September 2023, Song-Ming Tang, L5126 (HKAS 150784).

##### Notes.

Following the morphological analyses, *Lyophyllum pseudorrhizum* was placed in *L. fumosum* group; *L. fumosum*, *L. deqinense*, and *L. pseudorrhizum* are similar in that their pileus are all greyish-brown to greyish-orange in colour, their lamellae are all white to greyish-white, and they are all caespitose. However, *L. fumosum* differs from *L. pseudorrhizum* by its larger pileus (2–4.5 cm vs 1.2–2.8 cm wide), which is initially dark to light grey-brown, covered with dark greyish tufted squamules when young, becoming smooth and often with watery spots in age, and lacks the distinctive orange-toned floccose surface of *L. pseudorrhizum*. Furthermore, *L. fumosum* possesses a stipe that is often aggregated at the base but lacks the well-developed pseudorrhiza characteristic of *L. pseudorrhizum*; microscopically, *L. fumosum* is reported to lack pleurocystidia and cheilocystidia, whereas *L. pseudorrhizum* possesses both types of cystidia (Fries 1821; Sesli et al. 2015). *Lyophyllum deqinense* was originally described in China; it has a larger pileus (4–6 cm vs 1.2–2.8 cm wide), which is light to dark greyish orange; it lacks the aggregated pseudorrhiza; microscopically, *L. deqinense* is characterised by larger, subglobose to globose basidiospores (av. 6.4 × 6.1 µm vs 5.2 × 4.8 µm) and a complete absence of pleurocystidia and cheilocystidia (Ma et al. 2023).

In the phylogenetic tree (Fig. 1), *L. pseudorrhizum* forms a sister clade to *L. pallidofumosum*. However, *L. pallidofumosum* has a greyish orange pileus, margin with waves when old, stipe cylindrical, slightly enlarged towards base (30–80 × 8–15 mm vs 18–68 × 3–6 mm), solitary, gregarious or subcaespitose on the ground; microscopically, *L. pallidofumosum* absence of pleurocystidia and cheilocystidia (Ma et al. 2022); and ITS sequence differences between *L. pseudorrhizum* (HKAS 150783) and *L. pallidofumosum* (YAAS M6629) were 2.26% (12/533).

#### Lyophyllum hemigaleatum

Taxon classificationFungiAgaricalesLyophyllaceae

R.Y. Li, S.M. Tang sp. nov.

27B4BCE7-0651-5E98-BCF5-6AECC61B0694

Fungal Names: FN 573142

Figs 2D, 5, 6

##### Chinese name.

盔盖离褶伞 (Kui Gai Li Zhe San).

**Figure 5.**
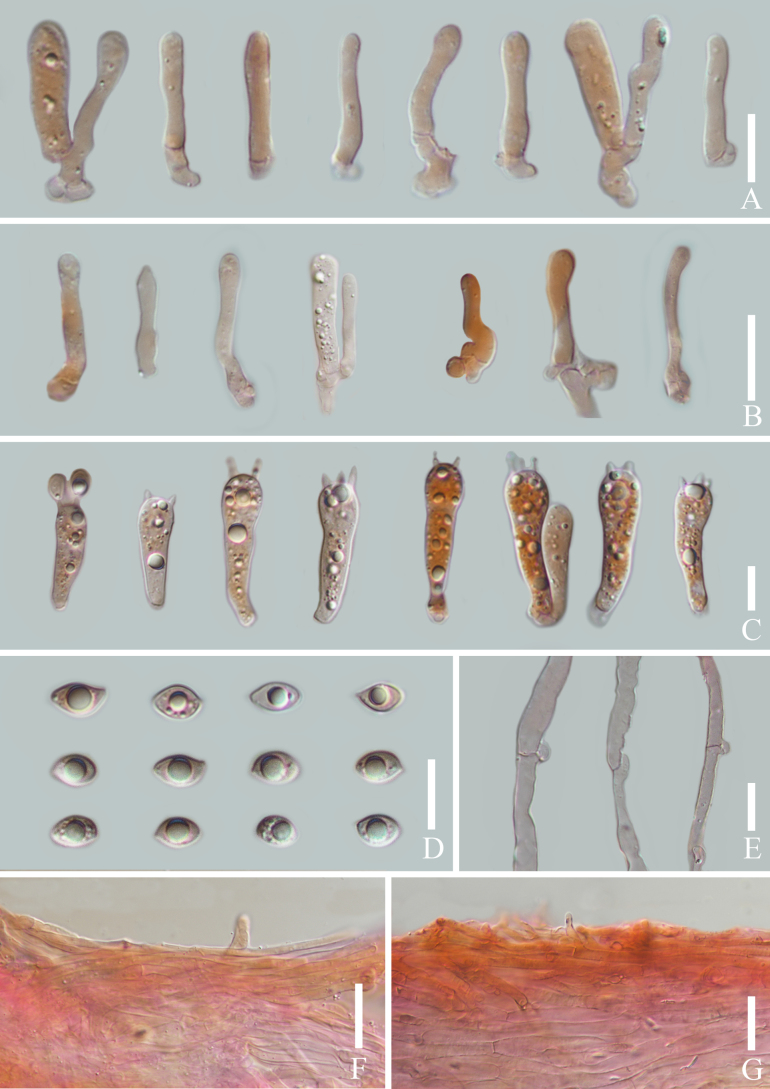
*Lyophyllum hemigaleatum* (HKAS 150782, holotype). **A**. Pleurocystidia; **B**. Cheilocystidia; **C**. Basidia; **D**. Basidiospores; **E**. Clamp connections of the hymenophoral trama; **F**. Stipitipellis; **G**. Pileipellis. Scale bars: 10 μm (**A–E**); 20 μm (**F, G**).

**Figure 6.**
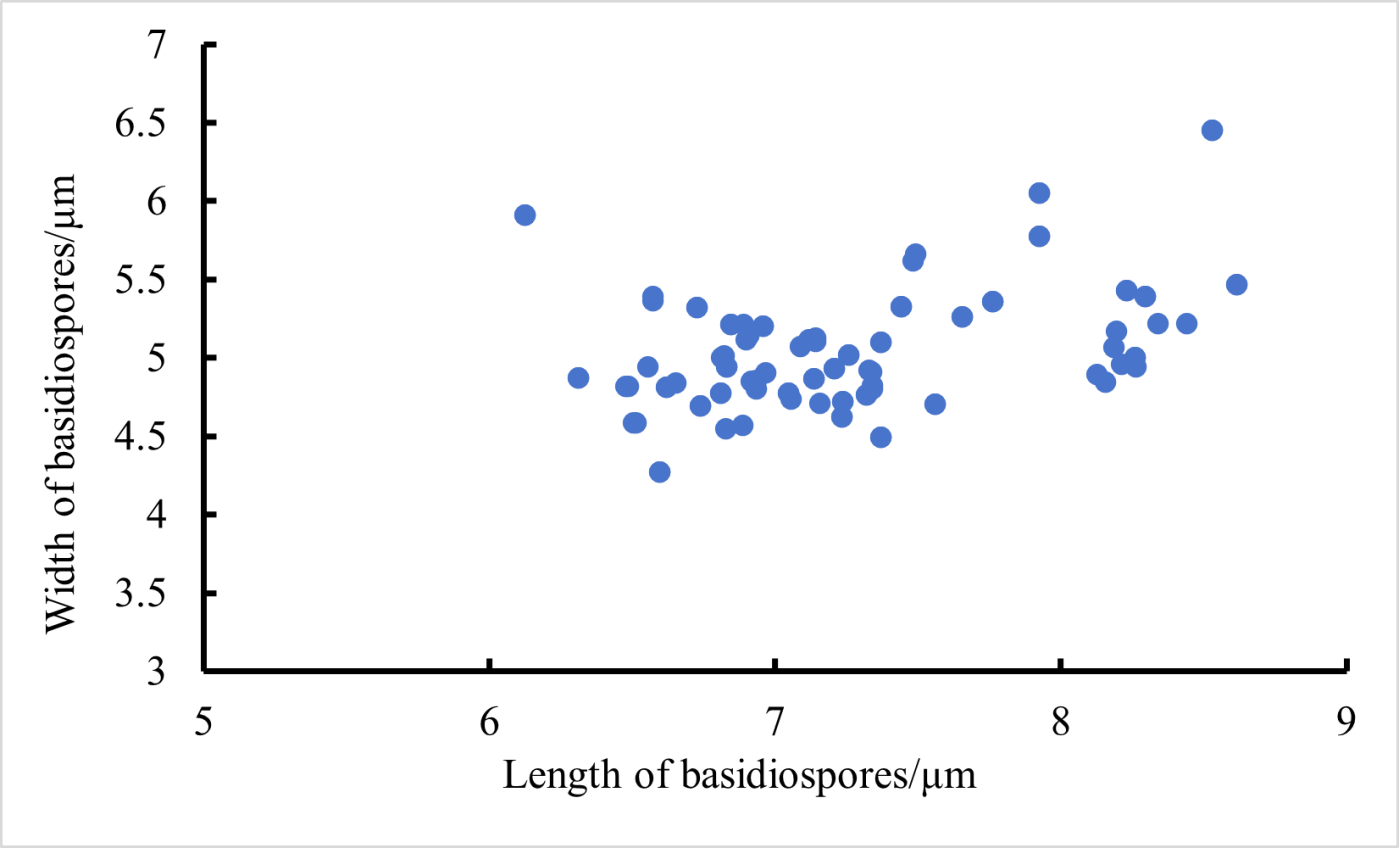
Basidiospore scatter plot of *Lyophyllum hemigaleatum*.

##### Etymology.

hemigaleatum (half-helmeted), describing the distinctive hemispherical pileus resembling a warrior’s helmet.

##### Holotype.

China, Yunnan Province, Dali City, Jianchuan County, elev. 2,200 m, 7 September 2024, Rui-Yu Li, 9714 (HKAS 150782!).

##### Description.

***Pileus*** 1.4–1.8 cm, hemispherical when young, inflexed of margin; becoming convex when mature, sometimes slightly depressed in the centre, dry, glabrous, slightly desaturated orange (#d0a87b) on centre, dark at the margin, dark moderate orange (#75533a); pileus context white (#ffffff), 0.1–0.2 cm thick, fleshy, thinner toward its margin, slightly desaturated orange (#b99b7e) when exposed or injured. ***Lamellae*** moderately close together, adnate to emarginate, broad, white (#ffffff), 2–4 tiers, 0.3–1.0 cm wide, edge even or entire to undulate. ***Stipe*** 2.8–4.2 cm long, 0.3–0.4 cm thick, cylindrical, equal or attenuate at the base, greyish yellow (#b6b09a); stipe context changing to slightly desaturated orange (#b99b7e) when injured. The odor and taste were not distinctive.

***Basidiospores*** [100/2/2] (6.1–) 6.5–8.3 (–8.6) × (4.3–) 4.5–5.8 (–6.5) μm, (N = 100, Q = 1.10–1.77, Q_m_ = 1.45 ± 0.15), av. 7.36 ± 0.65 × 5.10 ± 0.44 μm, in face view, the spores are broadly to subbroadly fusiform, while in lateral view, they exhibit a profile dominated by a suprahilar depression, hyaline, smooth, basidiospores scatter plot, see Fig. 6. ***Basidia*** 21–35 × 7–10 μm (N = 20), av. 28.5 ± 3.3 × 8.5 ± 0.9 μm, mostly 4-spored, rarely 2-spored, sterigmata 1.7–5.3 μm long, clavate to narrowly clavate, sometimes with basal clamp connections. ***Subhymenium*** consisting of thin and hyaline elements, 13–22 μm wide, with 2–3 layers of ovoid, subglobose, broadly fusiform, 1.7–11.0 × 1.4–4.1 μm, av. 5.1 ± 2.3 × 2.9 ± 0.8 μm. ***Hymenophoral trama*** regular, made up of inflated hyaline hyphae, 120–170 μm wide, some with clamp connections, narrowly cylindrical hyphal elements, 4–20 μm wide. ***Pleurocystidia*** 14–24 × 3–5 μm, av. 18.8 ± 2.3 × 3.8 ± 0.4 μm, narrowly cylindrical, narrowly clavate or flexuose, thin-walled. ***Cheilocystidia*** 8–24 × 2–5 μm, av. 17.7 ± 3.4 × 3.7 ± 0.6 μm, narrowly cylindrical, narrowly clavate or flexuose, thin-walled, occasionally mucronate on the apex. ***Pileipellis*** a subcutis composed of 2–7 μm wide hyphae, almost cylindrical, abundant pileocystidia present on the pileus, 5–31 × 2–5 µm, av. 19.8 ± 10.3 × 3.5 ± 0.6 µm, fusiform, narrowly cylindrical or flexuose, irregular, with mucronate apex, thin-walled, some with clamp connections. ***Stipitipellis*** composed of appressed, parallel, thin-walled, smooth, 2–5 µm wide hyphae, stipe surface bears abundant easily detachable pigment cells. ***Clamp connections*** are present at some septa.

##### Habitat.

Single to scattered on soil in coniferous forest, from August to October.

##### Additional species examined.

China, Yunnan Province, Dali City, Jianchuan County, elev. 2,180 m, 7 September 2024, Rui-Yu Li, T86 (HKAS 150788, paratype).

##### Notes.

Compared with the species with orange to brown pileus and a slender stipe, *L. edulis*, *L. aemiliae* Consiglio, and *L. lixivium* are similar to the new species. *Lyophyllum edulis* has a larger pileus (3–8 cm vs 1.4–1.8 cm), globose to subglobose basidiospores (av. 5.81 × 5.47 μm, Q_m_ = 1.11 vs 7.36 × 5.10 μm, Q_m_ = 1.45); additionally, *L. hemigaleatum* possesses abundant cystidia on the pileus surface, a feature not reported in *L. edulis* (Tang et al. 2025a). *Lyophyllum aemiliae*, originally described from Italy, is distinguished by a pileus with a wavy-lobed margin, slight streaking and irregular blackish-brown patches, in addition to a parallel pileipellis (Melis et al. 2023). *Lyophyllum lixivium* can be distinguished by its pileus developing a distinct umbo at maturity, and its relatively long stipe (10–14 cm vs 2.8–4.2 cm) with a slightly swollen base; basidiospores are broadly lacrimoid and relatively small (4.4–6.5 × 3.7–5.8 μm vs 6.5–8.3 × 4.5–5.8 μm), pileipellis is composed of parallel hyphae (Lyu et al. 2024).

In multi-locus phylogenetic analysis, the new species is closely related to *Lyophyllum semitale* and *L. deliberatum* (Britzelm.) Kreisel. *Lyophyllum semitale* was originally described from Sweden and has a leaden-grey pileus that is larger than *L. hemigaleatum* (2.5 cm vs 1.4–1.8 cm) and a white stipe; this species is lignicolous and grows saprophytically on decaying wood (Kühner 1938). In contrast, *L. hemigaleatum* bears orange-toned basidiomata and occurs terrestrially on mineral soil within coniferous forests, the ITS sequence differences between *L. hemigaleatum* (HKAS 150782, type) and *L. semitale* (IE-BSG-HC 85/13) were 3.03% (16/528). *Lyophyllum subalpinarum* from China has a greyish-yellow to brown pileus and is darker at the centre, and ITS sequence differences between *L. hemigaleatum* (HKAS 150782) and *L. subalpinarum* (HMJAU 63447) were 2.46% (13/529). *Lyophyllum deliberatum* was originally described in Europe; the pileus is brown to olive, and the stipe enlarged toward the base, and ITS sequence differences between *L. hemigaleatum* (HKAS 150782) and *L. deliberatum* (V. 15032) were 3.02% (16/529).

## Discussion

The phylogenetic tree was constructed following the methodology of Hofstetter et al. (2002), revealing that *L. hemigaleatum* forms a sister clade with *L. semitale* (IE-BSG-HC 85/13) from Switzerland. Notably, two specimens identified as *L. semitale* were placed in distinct positions on the phylogenetic tree (IE-BSG-HC 85/13 from Switzerland and EL 187-09 from Sweden). This phylogenetic divergence indicates that these specimens may represent separate species, necessitating a detailed comparison with the original description of *L. semitale*. The type locality of *L. semitale* is Sweden, and the species has a saprotrophic habit on wood, featuring a leaden-grey pileus that is larger than that of *L. hemigaleatum* (2.5 cm vs. 1.4–1.8 cm) and a white stipe. In contrast, *L. hemigaleatum* bears orange-toned basidiomata and occurs terrestrially on mineral soil within coniferous forests. These morphological features and ecological preferences differ markedly from those observed in the proposed new species, *L. hemigaleatum*. Although the original description of *L. semitale* does not include illustrations, morphological comparisons based on the recorded traits, together with phylogenetic analyses that clearly separate *L. hemigaleatum* from *L. semitale* EL 187-09 from Sweden, provide strong evidence for the distinction between these taxa. Therefore, *L. hemigaleatum* is tentatively identified as an independent species.

The *Lyophyllum decastes* complex is a taxonomically challenging group due to the high morphological similarity among its constituent species. As reported by Larsson and Sundberg (2011), the complex can be divided into four major lineages: the *L. shimeji* group, *L. decastes* group, *L. fumosum* group, and *L. loricatum* group. However, species in these groups are notoriously difficult to distinguish solely on the basis of traditional morphological features. Our findings further support this conclusion, as macroscopic characters of the sampled specimens showed substantial overlap, rendering them virtually indistinguishable. Earlier studies have noted the inadequacy of morphology for resolving species boundaries within this complex. Moncalvo and Clémençon (1992) suggested that reliable differentiation requires a combination of subtle characteristics, specifically variations in pileus, stipe, and lamellae colour. The reliance on such narrowly defined traits highlights the inherent limitations of phenotypic identification and the persistent risk of misclassification.

Attempts to use physiological or cultural characteristics have also yielded limited taxonomic resolution (Moncalvo et al. 1990). For instance, Moncalvo et al. (1993) and Arana-Gabriel et al. (2018) examined growth rates and biomass production of *Lyophyllum* strains in both solid and liquid media. Although these studies provided comparative data on strain growth behaviour, they did not identify consistent diagnostic traits sufficient for distinguishing species within the complex. Thus, physiological markers, like morphology, appear inadequate for resolving species boundaries.

Collectively, these findings emphasize that neither macroscopic characters nor growth-based physiological traits provide a reliable basis for species delimitation in the *L. decastes* complex. This underscores the need to integrate molecular data for accurate taxonomic resolution and highlights the importance of multilocus phylogenetic analyses for clarifying species diversity within this group (Li et al. 2019).

To identify morphological characteristics that distinguish specific groups, this study compiled a table of morphological traits (Table 2) for species within the *Lyophyllum decastes* complex, based on an extensive literature review. The results indicate that species in the *L. shimeji* group are predominantly characterised by a grey-toned pileus, ranging from grey-brown to grey-black, with the pileus surface typically covered in appressed brown floccus. The *L. fumosum* group is distinguished by a mainly white pileus, often appearing pale white or yellowish, with a smooth surface. The *L. decastes* group is defined by a grey pileus lacking brown or yellow pigmentation. The *L. loricatum* group has not been recorded in China.

**Table 2.** Morphological characteristics of species in the *Lyophyllum decastes* complex.

**Taxa**	**Pileus colour**	**Stipe colour**	**Lamellae colour**	**Pseudorrhiza**	**Reference**
*L. aurantiacum*	Greyish orange	Greyish orange	White	Without	Li et al. 2025
*L. decastes*	Light grey or greyish-black	White to pale, with a greyish to brown base	White	Without	Qi et al. 2007
*L. deqinense*	Light greyish orange to dark greyish orange	White	White	Without	Ma et al. 2023
*L. edulis*	Dark greyish orange or soft orange	Greyish orange	White	Without	Tang et al. 2025a
*L. fumosum*	Light grey to greyish	Whitish grey to grey	White	Without	Orton 1960
*L. heimogu*	Dark grey olive	Yellowish brown	White	Without	Li et al. 2023
*L. herrerae*	Greyish brown to yellowish brown	Cream to pale greyish yellow	Cream to pale yellow	Without	Arana-Gabriel et al. 2025
*L. littoralis*	Mottled grey to brownish grey colour	White to grey	White to pale yellow grey	Without	Contu 1998
*L. loricatum*	Lead grey to dark brown	Pale brown to dark brown	Whitish pale cream, yellowing with age	Without	Fries 1838
*L. pallidofumosum*	Desaturated orange to greyish orange	White	White to light greyish orange	Without	Ma et al. 2022
*L. pseudorrhizum*	Greyish orange, dark greyish orange	Light greyish yellow to slightly desaturated orange	White	Occurs	This study
*L. shimeji*	Dark orange	Greyish yellow	White	Without	Tang et al. 2025a
*L. subdecastes*	Brown or greyish-red	White	White	Without	Wei et al. 2023
*L. yiqunyang*	Olive-grey	White to pale grey	White	Without	Li et al. 2023

Despite these group-level patterns, species within the *L. decastes* complex exhibit considerable morphological similarity and plasticity. Although multi-locus phylogenetic analyses have significantly enhanced species delimitation, there remains a critical need to identify novel and stable diagnostic morphological characters to enable more accurate identification and differentiation within this complex.

The *Lyophyllum fumosum* group was originally established based on *L. fumosum*, described from Europe and typically occurring in coniferous or deciduous forests with grassy undergrowth (Fries 1821; Larsson and Sundberg 2011). In the present study, two new species were identified in Yunnan Province, southwestern China. Based on morphological and ecological characteristics, as well as moderate support from phylogenetic analyses, *L. pseudorrhizum* is classified within the *L. fumosum* group, thereby expanding the taxonomic circumscription of this group. However, a re-examination of published descriptions indicates that the basidiomata of true *L. fumosum* grow gregariously or caespitose and lack a distinct sclerotium-like basal structure, whereas those of *L. pseudorrhizum* form dense clusters with a well-developed, tuber-like base. Consequently, *L. pseudorrhizum* is recognized as a new species.

## Supplementary Material

XML Treatment for Lyophyllum pseudorrhizum

XML Treatment for Lyophyllum hemigaleatum

## Additional information

### Artificial Intelligence (AI) use

The authors accept full responsibility for the content of the manuscript, including the disclosure of any use of AI.

No AI tools were used in the preparation of this manuscript.

### Author ORCIDs

R.-Y. Li https://orcid.org/0009-0008-2136-9460

Y. Luo https://orcid.org/0009-0001-0244-1462

D.-F. Zhao https://orcid.org/0009-0000-4251-3988

Y.-T. Li https://orcid.org/0009-0006-7153-751X

H.-B. Song https://orcid.org/0000-0002-7440-5444

H.-W. Shen https://orcid.org/0000-0003-2508-1970

Z.-L. Luo https://orcid.org/0000-0001-7307-4885

S.-M. Tang https://orcid.org/0000-0003-0665-6900

### Data availability

All of the data that support the findings of this study are available in the main text.
